# Establishment and validation of an AI-aid method in the diagnosis of myocardial perfusion imaging

**DOI:** 10.1186/s12880-023-01037-y

**Published:** 2023-06-16

**Authors:** Ruyi Zhang, Peng Wang, Yanzhu Bian, Yan Fan, Jianming Li, Xuehui Liu, Jie Shen, Yujing Hu, Xianghe Liao, He Wang, Chengyu Song, Wangxiao Li, Xiaojie Wang, Momo Sun, Jianping Zhang, Miao Wang, Shen Wang, Yiming Shen, Xuemei Zhang, Qiang Jia, Jian Tan, Ning Li, Sen Wang, Lingyun Xu, Weiming Wu, Wei Zhang, Zhaowei Meng

**Affiliations:** 1 Department of Nuclear Medicine, Tianjin Medical University General Hospital, Anshan Road No. 154, Heping District, Tianjin, China 300052; 2 Department of Nuclear Medicine, Hebei General Hospital, Shijiazhuang, China; 3 Department of Nuclear Medicine, Peking University First Hospital, Beijing, China; 4 Department of Nuclear Medicine, Teda International Cardiovascular Hospital, Tianjin, China; 5 Department of Nuclear Medicine, Tianjin Third Central Hospital, Tianjin, China; 6 Department of Nuclear Medicine, Tianjin First Central Hospital, Tianjin, China; 7 School of Microelectronics, Tianjin University, Weijin Road No. 92, Nankai District, Tianjin, China 300072; 8 Department of Nuclear Medicine, The Affiliated Cancer Hospital of Zhengzhou University & Henan Cancer Hospital, Zhengzhou, China

**Keywords:** Artificial intelligence (AI), Machine learning, Coronary artery disease (CAD), Myocardial perfusion imaging (MPI), SPECT/CT

## Abstract

**Background:**

This study aimed to develop and validate an AI (artificial intelligence)-aid method in myocardial perfusion imaging (MPI) to differentiate ischemia in coronary artery disease.

**Methods:**

We retrospectively selected 599 patients who had received gated-MPI protocol. Images were acquired using hybrid SPECT-CT systems. A training set was used to train and develop the neural network and a validation set was used to test the predictive ability of the neural network. We used a learning technique named “YOLO” to carry out the training process. We compared the predictive accuracy of AI with that of physician interpreters (beginner, inexperienced, and experienced interpreters).

**Results:**

Training performance showed that the accuracy ranged from 66.20% to 94.64%, the recall rate ranged from 76.96% to 98.76%, and the average precision ranged from 80.17% to 98.15%. In the ROC analysis of the validation set, the sensitivity range was 88.9 ~ 93.8%, the specificity range was 93.0 ~ 97.6%, and the AUC range was 94.1 ~ 96.1%. In the comparison between AI and different interpreters, AI outperformed the other interpreters (most *P*-value < 0.05).

**Conclusion:**

The AI system of our study showed excellent predictive accuracy in the diagnosis of MPI protocols, and therefore might be potentially helpful to aid radiologists in clinical practice and develop more sophisticated models.

**Supplementary Information:**

The online version contains supplementary material available at 10.1186/s12880-023-01037-y.

## Background

Coronary artery disease (CAD) is one of the leading causes of morbidity and mortality throughout the world [1]. According to the report released by the American Heart Association (AHA) in 2016, over 15.5 million people above 20 years of age suffer from CAD in the United States [2]. Currently, imaging methods regarding CAD diagnosis include electrocardiography [3], invasive coronary angiography (ICA) or non-invasive tomographic coronary angiography (CTCA) [4, 5], myocardial perfusion imaging (MPI) [6], ultrasonography [7], etc. All these methods have been proved to be effective. Among them, MPI with single-photon emission computer tomography (SPECT) is a well-established non-invasive test in terms of the evaluation of ischemia, scar, left ventricular volumes, and ejection fraction, by directly reflecting the tracer uptake of the heart [8]. With advances during the past few years, MPI has certainly evolved from a diagnostic test of high accuracy for the detection of CAD (reportedly with mean sensitivity and specificity of 90% and 75%, respectively) to an essential tool for risk stratification [9, 10].

Traditionally, the interpretation of medical imaging requires one’s sufficient knowledge in the medical-related domains [11], which needs decades of training. Clinically, this interpretation process can also be time-consuming. Simultaneously, patients have been demanding faster and more personalized care [12, 13]. The resultant shortage of physicians and the requirement for efficiency emerged thereafter. Inspiringly, artificial intelligence (AI, or machine learning) is poised to influence nearly every aspect of human life, especially in the medical field. These years, the combination of AI and medical imaging has been remaining as a hot topic and there is presently sufficient evidence demonstrating its practicability (e.g. AI with ultrasound, computed tomography, magnetic resonance imaging, or histopathology) [14–17]. However, the application of AI in nuclear medicine imaging and, in particular, MPI can be troublesome. Firstly, MPI images are multi-slices rather than planer images and the interpretation is mostly based on the information seen in multiple slices [18]. Secondly, the interpretation also relies on the agreement of images from various axes (i.e., short-axis, horizontal long-axis, vertical long-axis, and polar map) [19, 20]. These issues might hamper the establishment of an AI-aid diagnostic system in MPI. According to our limited knowledge, approaches to these problems are yet scarce, and we herein introduce an AI-aid method to detect ischemic abnormalities in MPI.

## Materials and methods

### Sample collection

We retrospectively selected 599 patients from the participating medical centers, and among them, 379 (63.27%) cases were males and 220 (36.73%) cases were females. All patients had received gated-MPI protocol using ^99m^Tc-sestamibi. Images were acquired using four hybrid SPECT-CT systems including (Discovery NM/CT 670 CZT, GE Healthcare; Discovery NM 530c, GE Healthcare; Symbia T16, Siemens Corp.). Acquisition parameters were as follows:

1. Discovery NM/CT 670 CZT: 64 × 64 matrix size; 1.3 zoom; 30 secs per view (30 views in total); 140 keV ± 10% main energy window; 120 kVp CT tube voltage; 20 mA tube current; 1.25 mm slice thickness.
2. Discovery NM 530c: 64 × 64 matrix size; no zoom; 30 secs per frame (48 frames in total); 140 keV ± 10% main energy window; without CT acquisition.
3. Symbia T16: 64 × 64 matrix size; 1.45 zoom; 16 secs per view (32 views in total); 140 keV ± 10% main energy window; without CT acquisition.

Reconstruction parameters were as follows:

- All images were reconstructed using FBP algorithm with Butterworth filter (critical frequency: 0.45 ~ 0.50). The correction methods used in the reconstruction process included CT-based AC (Discovery NM/CT 670 CZT), and dual-energy-window technique-based SC.
- Individuals’ MPI images were pulled out for preparation. The mean diagnostic age was 59.14-year-old with a standard deviation of ± 11.61. All patients had or were suspected to have CAD-related presentations prior to the SPECT scan. MPI images consisted of three conventional axes: short-axis (SA, 13,267 slices), horizontal long-axis (HLA, 11,465 slices), and vertical long-axis (VLA, 11,676 slices). Patients were divided into two subsets including a training set and a validation set. By doing so, firstly, images of all patients were indexed in sequence and therefore 13,267 slices of SA, 11,465 slices of HLA, and 11,676 slices of VLA were totally indexed. Secondly, with these index numbers, we then separated these images randomly before training using Python software (version 3.7.3). The training set was then used to train and develop the following neural network accounting for 70% (each axis), and the validation set was used to test the predictive ability of the neural network accounting for 30% (each axis).

### Machine learning network selection

Machine learning strategies can be generally split into either unsupervised or supervised learning. The main scope of unsupervised learning is to discover underlying structure or relationships among variables in a dataset, whereas supervised learning normally requires the classification of one or more categories or outcomes [21]. Due to the particularity of medical images which often requires the evaluation of multiple categories, in this study, we selected the supervised learning method-regional deep learning technique, an ROI (region of interest)-based conventional neural network named YOLO (you only look once, version 3), [22] to complete the training. The YOLO algorithm was composed of four main stages: preprocessing of the tagged images, feature extraction utilizing deep convolutional networks (training), lesion detection with confidence (calculating), and finally lesion classification using fully connected neural networks (FC-NNs, output) [23]. Machine learning network was implemented using Python software.

### Tagging method for CAD-suspected lesions

**Fig. 1:**
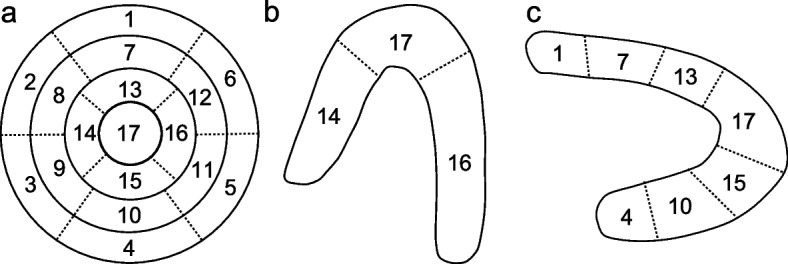
Standardized myocardial segmentation and nomenclature for tomographic imaging of the heart. **a** short-axis; **b** horizontal long-axis; **c** vertical long-axis; 1, basal anterior; 2, basal anteroseptal; 3, basal inferoseptal; 4, basal inferior; 5, basal inferolateral; 6, basal anterolateral; 7, mid anterior; 8, mid anteroseptal; 9, mid inferoseptal; 10, mid inferior; 11, mid inferolateral; 12, mid anterolateral; 13, apical anterior; 14, apical septal; 15, apical inferior; 16, apical lateral; 17, apex

Images were tagged in accordance with the standardized myocardial segmentation and nomenclature for tomographic imaging of heart proposed by the ATA in 2002 [24]. Therefore, a total of 17 tags (from the basal to the apical) were applied in the tagging process (Fig. 1). If a lesion was identified to exist in the apex wall, we delineate that area with a tag “17” in all three axes (i.e. SA, HLA, and VLA). Accordingly, if a lesion was detected in the non-apical segment like the mid inferior wall, we give that area a tag “10” both in SA and VLA. In the input process of training, images of three axes were sent to the model separately and therefore a total of three sub-models were trained and then incorporated into one model. When detecting ischemia, these sub-models will give three independent results based on the ischemic area. A program named “labelme” under the Anaconda environment was used in this tagging step.

### Statistical analysis

The age distribution of patients was tested using the Kolmogorov–Smirnov method. The lesion distribution of the 17 segments among SA, HLA, and VLA was tested using the Chi-square test. A *P*-value < 0.05 was considered to be statistically significant.

### Validation of the neural network

To test the training performance of the neural network, we calculated the average precision and recall rate. Additionally, in order to test its general clinical accuracy, ROC analysis was applied using the validation set. The gold standard was set as the diagnostic report made by an agreement of two experienced interpreters with at least 30 years of high-volume medical-related background (expert). We also randomly selected 100 slices (random validation set) of each axis and compared the statistical differences of sensitivity, specificity among AI, beginner (with 1 week of training, intern), inexperienced interpreter (with 5 years of medical-related background, resident physician), and experienced interpreter, by using McNemar’s test [25]. To test the consistency between AI and the gold standard, a consistency check was also performed by calculating Cohen’s Kappa coefficients. Lastly, to evaluate the diagnostic speed of AI, we performed a time consumption analysis between AI and experienced interpreters based on 60 patients selected from the validation set. We compared the distribution and statistical differences in terms of time consumption between them. All statistics were derived by using SPSS 23.0 (IBM, USA) and a *P*-value < 0.05 was considered to be statistically significant.

## Results

### Patient and lesion distribution

**Fig. 2:**
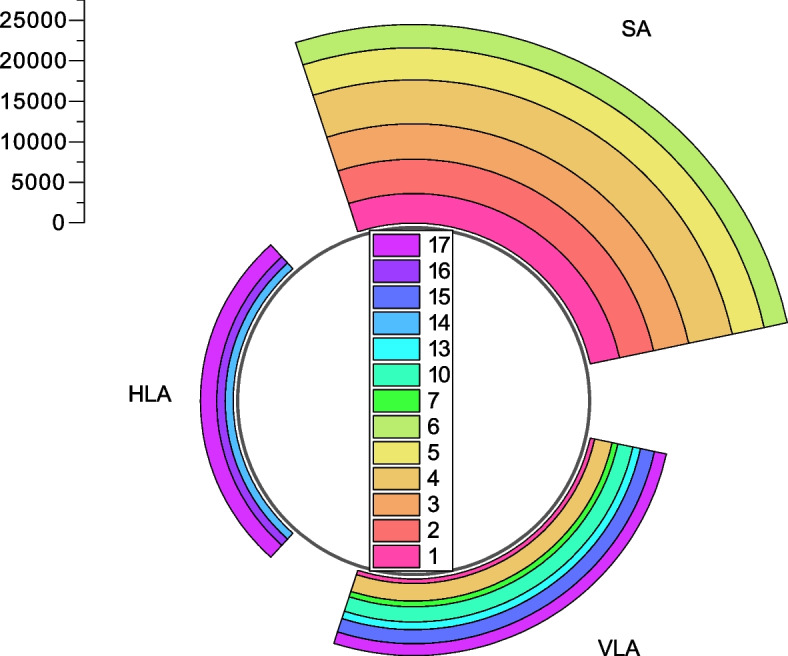
Lesion distribution among three axes. SA, Short axis; HLA, Horizontal long axis; VLA, Vertical long axis; 1, Anterior for SA (Basal anterior for VLA); 2, Anteroseptal; 3, Interseptal; 4, Inferior for SA (Basal inferior for VLA); 5, Inferolateral; 6, Anterolateral; 7, Mid anterior; 10, Mid inferior; 13, Apical anterior, 14, Septal; 15, Apical inferior; 16, Lateral; 17, Apical

Overall, there was a normal distribution in both the male group and female group (all *P*-value > 0.05). Also, the diagnostic age of females was older than that of males (distribution peak at around 65-year-old for the female group and around 55-year-old for the male group). As shown in Table 1 and Fig. 2, SA accounted for the majority of lesions among all three axes (24,539 for SA vs. 3,978 for HLA vs. 9,479 for VLA). Additionally, for each axis, statistically significant differences were derived among different segments (all *P*-value < 0.001). Among different segments, segment 4 (inferior) accounted for the majority in SA (22.20%), segment 17 (apical) accounted for the majority in HLA (49.52%), and segment 4 accounted for the majority in VLA (22.73%) (Table 1 and Fig. 2). In comparison, segment 6 (anterolateral) accounted for the minority in SA (11.79%), segment 14 (septal) accounted for the minority in HLA (24.46%), and segment 1 accounted for the minority in VLA (6.08%) (Table 1 and Fig. 2).

**Table 1:** Lesion distribution of the 17 segments among three axes

	SA n (%)	HLA n (%)	VLA n (%)	Total n (%)
1	3,668 (14.95)	-	576 (6.08)	4,244 (11.17)
2	4,221 (17.20)	-	-	4,221 (11.11)
3	4,376 (17.83)	-	-	4,376 (11.52)
4	5,447 (22.20)	-	2,155 (22.73)	7,602 (20.01)
5	3,935 (16.04)	-	-	3,935 (10.36)
6	2,892 (11.79)	-	-	2,892 (7.61)
7	-	-	713 (7.52)	713 (1.88)
10	-	-	1,863 (19.65)	1,863 (4.90)
13	-	-	938 (9.90)	938 (2.47)
14	-	973 (24.46)	-	973 (2.56)
15	-	-	1,759 (18.56)	1,759 (4.63)
16	-	1,035 (26.02)	-	1,035 (2.72)
17	-	1,970 (49.52)	1,475 (15.56)	3,445 (9.07)
Total	24,539 (100.00)	3,978 (100.00)	9,479 (100.00)	37,996 (100.00)
*P*-value	< 0.001	< 0.001	< 0.001	< 0.001

*SA* Short axis, *HLA* Horizontal long axis, *VLA* Vertical long axis, 1 Anterior for SA (Basal anterior for VL), 2 Anteroseptal, 3 Interseptal, 4 Inferior for SA (Basal inferior for VL), 5 Inferolateral, 6 Anterolateral, 7 Mid anterior, 10 Mid inferior, 13 Apical anterior, 14 Septal, 15 Apical inferior, 16 Lateral, 17 Apical

### Metrics of accuracy, recall, and precision in the training set

In SA, accuracy ranged from 74.37% to 84.73%, recall rate ranged from 94.04% to 98.76%, and average accuracy ranged from 90.04% to 98.15% (Table 2). The accuracy, recall rate, and average precision of segment 4 were larger than those of other segments (84.73%, 98.76%, and 98.15%, respectively).

**Table 2:** Training metrics of the short axis

	Accuracy (%)	Recall (%)	Average precision (%)
1	78.78	98.01	96.88
2	77.67	96.26	94.64
3	81.37	95.31	93.16
4	84.73	98.76	98.15
5	74.37	96.10	93.78
6	74.73	94.04	90.04

1 Anterior, 2 Anteroseptal, 3 Interseptal, 4 Inferior, 5 Inferolateral, 6 Anterolateral

In HLA, the range of accuracy was 66.20% ~ 79.32%, the range of recall rate was 88.50% ~ 92.77%, and the range of average accuracy was 81.90% ~ 90.34% (Table 3). Among segments 14, 17, and 16, segment 17 had the largest accuracy (79.32%), recall rate (92.77%), and average precision (90.34%), whereas segment 14 had the smallest numbers (66.20% for accuracy, 88.50% for recall rate, and 81.90% for average precision).

**Table 3:** Training metrics of the horizontal long axis

	Accuracy (%)	Recall (%)	Average precision (%)
14	66.20	88.50	81.90
17	79.32	92.77	90.34
16	70.83	88.85	87.02

14 Septal, 17 Apical, 16 Lateral

In VLA, the range of accuracy was 88.02% ~ 94.64%, the range of recall rate was 76.96% ~ 91.8%, and the range of average accuracy was 80.17% ~ 93.37 (Table 4). The greatest accuracy was found in segment 17 (94.64%), whereas the greatest recall rate and average precision were found in segment 1 (91.80% and 93.37%, respectively). In addition, the smallest accuracy was found in segment 15(87.91%) and the smallest recall rate and average precision were found in segment 13 (76.96% and 80.17%, respectively).

**Table 4:** Training metrics of the vertical long axis

	Accuracy (%)	Recall (%)	Average precision (%)
1	90.32	91.80	93.37
7	89.36	85.14	86.47
13	88.02	76.96	80.17
17	94.64	84.13	88.51
15	87.91	81.92	85.39
10	90.91	86.33	88.30
4	92.69	87.57	88.71

1 Basal anterior, 7 Mid anterior, 13 Apical anterior, 17 Apical, 15 Apical inferior, 10 Mid inferior; 4 Basal inferior

### Validation of the neural network

**Fig. 3:**
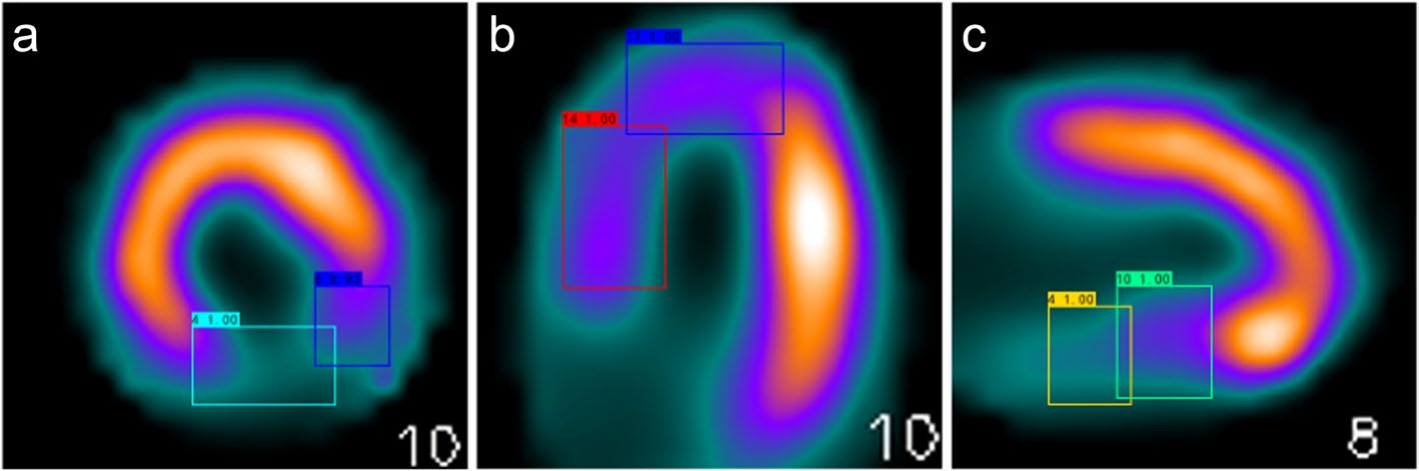
Lesions predicted by AI. **a** Short axis; **b** Horizontal long axis; **c** Vertical long axis

**Fig. 4:**
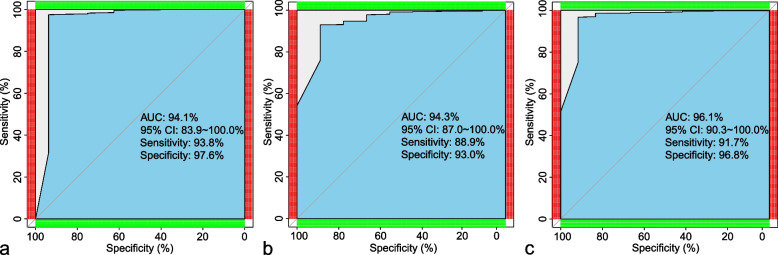
ROC analysis of three different axes. **a** Short axis; **b** Horizontal long axis; **c** Vertical long axis

Figure 3 was an example predicted by AI and segments of all lesions were identified accurately. In ROC analysis of the validation set, sensitivity, specificity, and AUC of SA were 93.8%, 97.6%, and 94.1%, respectively (Fig. 4a). The sensitivity, specificity, and AUC of HLA were 88.9%, 93.0%, and 94.3%, respectively (Fig. 4b). The sensitivity, specificity, and AUC of VLA were 91.7%, 96.8%, and 96.1%, respectively (Fig. 4c).

**Fig. 5:**
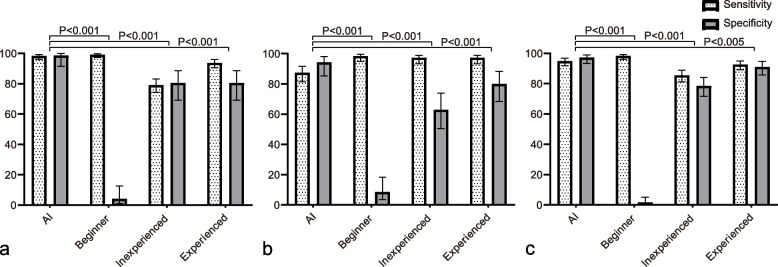
Comparison of both sensitivity and specificity among different interpreters. **a** short-axis; **b** horizontal long-axis; **c** vertical long-axis

As demonstrated in Fig. 5, when comparing both sensitivity and specificity with those of AI, all interpreters showed statistically significant differences in the random validation set (all *P*-value < 0.05). Table 5 showed the comparison of sensitivity among AI, beginner, inexperienced, and experienced interpreters in the random validation set. The beginner had the largest sensitivity in the SA (99.15%, 95% CI: 97.33 ~ 99.78%), HLA (98.35%, 95% CI: 94.87 ~ 99.57%), and VLA (98.30%, 95% CI: 96.14 ~ 99.31%). However, there was no statistically significant difference between the beginner and AI in SA. The sensitivity of the experienced interpreter was larger than that of AI in HLA and there was a statistically significant difference (*P*-value < 0.001), but lower than those of AI in both SA and VLA (*P*-value of SA < 0.05, *P*-value of VLA > 0.05). In comparison, the inexperienced interpreter had the lowest sensitivity among all interpreters in both SA and VLA and there were statistically significant differences when comparing with AI (*P*-value < 0.001). However, in terms of the HLA axis, it had the same sensitivity as the experienced interpreter.

**Table 5:** Comparison of sensitivity among AI, beginner, inexperienced, and experienced in the random validation set

	AI (%)	Beginner (%)	Inexperienced (%)	Experienced (%)
SA	98.30 (96.15 ~ 99.31)	99.15 (97.33 ~ 99.78)	79.04 (74.34 ~ 83.09) ††	93.77 (90.58 ~ 95.96) †
HLA	87.36 (81.44 ~ 91.66)	98.35 (94.87 ~ 99.57) ††	97.25 (93.36 ~ 98.98) ††	97.25 (93.36 ~ 98.98) ††
VLA	94.89 (91.89 ~ 96.85)	98.30 (96.14 ~ 99.31) †	85.51 (81.30 ~ 88.93) ††	92.61 (89.23 ~ 95.03)

^†^*P*-value < 0.05

^††^*P*-value < 0.001

() 95% confidence interval, *SA* Short axis, *HLA* Horizontal long axis, *VLA* Vertical long axis

As shown in Table 6, the beginner had the smallest specificities in all three axes (4.17% in SA, 8.57% in HLA, 1.66% in VLA), and there were statistically significant differences compared with AI (all *P*-value < 0.001). Conversely, AI had the largest specificities in SA (98.61%, 95% CI: 91.46 ~ 99.93%), HLA (94.29%, 95% CI: 85.27 ~ 98.15%), and VLA (97.24%, 95% CI: 93.33 ~ 98.98%).Although the inexperienced interpreter and the experienced interpreter had the same specificity in SA, it could be noticed that the specificities of the experienced interpreter were larger than those of the inexperienced interpreter in HLA and VLA. Additionally, the specificities of both the inexperienced and experienced interpreters had statistically significant differences (all *P*-value < 0.05).

**Table 6:** Comparison of specificity among AI, beginner, inexperienced, and experienced in the random validation set

	AI (%)	Beginner (%)	Inexperienced (%)	Experienced (%)
SA	98.61 (91.46 ~ 99.93)	4.17 (1.08 ~ 12.50) ††	80.56 (69.20 ~ 88.59) ††	80.56 (69.20 ~ 88.59) ††
HLA	94.29 (85.27 ~ 98.15)	8.57 (3.53 ~ 18.35) ††	62.86 (50.43 ~ 73.86) ††	80.00 (68.39 ~ 88.26) †
VLA	97.24 (93.33 ~ 98.98)	1.66 (0.43 ~ 5.16) ††	78.45 (71.61 ~ 84.06) ††	91.16 (85.80 ~ 94.70) †

^†^*P*-value < 0.05

^††^*P*-value < 0.001

() 95% confidence interval, *SA* Short axis, *HLA* Horizontal long axis, *VLA* Vertical long axis

Consistency check showed that AI had the best agreement with the gold standard in all three axes (Cohen’s Kappa coefficients: 0.943 for SA, 0.754 for HLA, and 0.905 for VLA, all *P*-value < 0.001, Table 7). Likewise, the beginner had the smallest agreement with the gold standard (Cohen’s Kappa coefficients: 0.052 for SA, 0.095 for HLA, and 0.013 for VLA; *P*-value of SA and HLA < 0.05, *P*-value of VLA > 0.05). Cohen’s Kappa coefficients of the experienced interpreter were smaller than those of AI but larger than those of the inexperienced interpreter (0.712 vs. 0.447 for SA, 0.804 vs. 0.662 for HLA, and 0.827 vs. 0.630 for VLA, all *P*-value < 0.001).

**Table 7:** Consistency check (diagnostic method vs. gold standard) of sensitivity and specificity in the random validation set

	Cohen’s Kappa			
	AI	Beginner	Inexperienced	Experienced
SA	0.943 ††	0.052 †	0.447 ††	0.712 ††
HLA	0.754 ††	0.095 †	0.662 ††	0.804 ††
VLA	0.905 ††	0.013	0.630 ††	0.827 ††

^†^< 0.05

^††^< 0.001

*SA* Short axis, *HLA* Horizontal long axis, *VLA* Vertical long axis

**Fig. 6:**
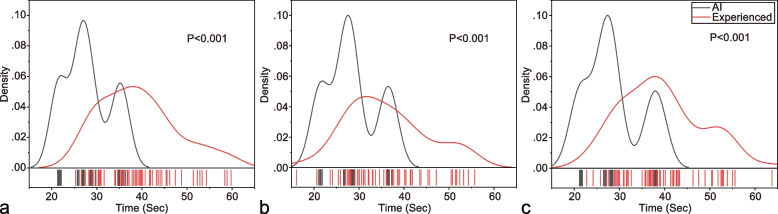
Comparison of time consumption distribution between AI and experienced interpreter. **a** short-axis; **b** horizontal long-axis; **c** vertical long-axis; statistical difference derived from Wilcoxon test

In the time consumption analysis of the selected dataset, it took AI 1673.23 s in SA, 1698.41 s in HLA, and 1715.08 s in VLA to generate diagnoses, whereas it took the experienced interpreter 2348.67 s in SA, 2162.89 s in HLA, and 2352.98 s in VLA to give final prognoses. Also, the average time consumption of AI per axis was much less compared with that of the experienced interpreter (Supplementary Table 1). Figure 6 shows that AI completed the detective process mostly between 20 and 40 s in three axes. However, those numbers ranged largely from 20 to 60 s for the experienced interpreter.

## Discussion

Due to the shortage of large-scale publicly available datasets containing SPECT images for the detection of CAD, the application of deep learning has not been thoroughly explored. This study described a clinical application of an AI-aid system with explainable predictions tested in a large, multicenter population with gated-MPI protocols. This system demonstrated significantly high predictive accuracy and clinical availability in all three axes.

Among all cardiovascular diseases, CAD is a major cause of morbidity and mortality among adults worldwide and brings a heavy burden for the patients and their families [26]. In our cohort, more male patients received gated-MPI protocols (63.27% males vs. 36.73% females). This might be explained by the hypothesis that women often present with atypical symptoms and therefore continue to delay seeking treatment [27]. On the whole, the diagnostic age of CAD in females was larger than that of males (distribution peak at around 65-year-old for females and around 55-year-old for males). The increased number of elderly female patients might need targeted care.

The 17-segment model of the left ventricle, as an optimally weighted approach for the visual interpretation of regional left ventricular abnormalities, has been widely used since it was proposed. In terms of the correspondence between left ventricular 17 myocardial segments and coronary arteries, segments 1, 2, 7, 8, 13, 14, and 17 are assigned to the left anterior descending coronary artery distribution. Segments 3, 4, 9, 10, and 15 are assigned to the right coronary artery when it is dominant. Segments 5, 6, 11, 12, and 16 generally are assigned to the left circumflex artery [28]. Based on the research of Juillière, Y., et al., the most common segments of ischemia could be segment 1(anterior), segment 4 (inferior), and segment 17 (apical) [29]. Our study also revealed a similar distribution (15.52% for segment 1, 20.01% for segment 4, 9.07% for segment 17). The higher abnormality ratio of segment 4 in our study might be explained by the higher involvement ratio of stenosis in the right coronary artery, compared with those of the left anterior descending coronary artery or the left circumflex artery. Likewise, in a clinical trial of 215 patients conducted by Nordlund, D., et al., 39%of patients had left anterior descending artery occlusion, 49% had right coronary artery occlusion, and 12% had left circumflex artery occlusion [30]. This was also consistent with the distribution of our analysis.

In SA, the training accuracy, recall rate, as well as average precision of segment 4 were larger than those of other segments (84.73%, 98.76%, and 98.15%, respectively, Table 2). In HLA, segment 17 had the largest accuracy (79.32%), recall rate (92.77%), and average precision (90.34%), compared with other segments (Table 3). Similarly, the greatest accuracy was found in segment 17 (94.64%) and the greatest recall rate and average precision were found in segment 1 of VLA (91.80% and 93.37%, respectively, Table 4). The higher accuracy seen in these segments might be contributed from the relatively larger number of lesions among these segments in the training set (segment 4 accounted for 22.20% in SA, segment 17 accounted for 49.52% in HLA, and 15.56% in VLA, Table 1). This indicated that a large number of lesions is essential for AI to extract enough features and subsequently increase the training accuracy of algorithm architecture. Some researchers also concluded that datasets with adequate sample size are one of the dominant factors in developing and training effective computer-aided diagnosis algorithms [31, 32].

Apostolopoulos, I.D., et al. [33] proposed a method for automatic classification of polar maps based on a neural network named VGG16. The proposed model achieved a sensitivity of about 75.00% and a specificity of about 73.43%. Arsanjani, R., et al. [34] introduced a Support Vector Machine (SVM) algorithm in their study to predict the detection of ≥ 70% coronary artery lesions and their research yielded both relatively good sensitivity (84%) and specificity (88%) during validation. Betancur, J., et al. [35] developed an automatically predictive model to identify obstructive heart disease using deep learning and also achieved a good accuracy (AUC: 0.76 ~ 0.80). However, these studies did not incorporate different axes of the heart from MPI images as targeted training.

The results of our work highlight the capabilities of deep learning for classification tasks of nuclear medicine imaging. On the validation set, sensitivity, specificity, as well as AUC of all axes were all above 90% except for the sensitivity of HLA. On the random validation set, AI outperformed the beginner, the inexperienced interpreter, as well as the experienced interpreter, since it achieved both relatively larger sensitivities and specificities in all three axes (most *P*-value < 0.05, Tables 5 and 6). In terms of the comparison with the experienced interpreter, the proposed AI-aid system yielded a relatively equivalent performance of sensitivity. On the whole, the beginner had the largest sensitivities in all three axes (98.30 ~ 99.15%, Table 5). However, specificities in these axes were extremely low (1.66 ~ 8.57%). This suggested that the beginner could identify most of the ischemic lesions, but it resulted from the price of a large amount of false-positive lesions. Clinically, this situation must be avoided. However, even with a short period of training, this situation was not observed with AI, and therefore, this again confirmed the fact that AI, if with a proper design, has the intrinsically efficient learning capability in the classification of medical imaging. It could also be noticed that AI also had the best agreement in all axes (Cohen’s Kappa coefficients: 0.943 for SA, 0.754 for HLA, and 0.905 for VLA). These results suggest that the YOLO network, though an endeavor in our study, can be used as a promising approach in nuclear medicine. Several studies also confirmed the availability of it in medical imaging, since it offers an excellent tradeoff between accuracy and efficiency [22, 36, 37].

There were three limitations to our study. First, since this was a retrospective study, acquisition parameters of different SPECT systems in different institutions had already been fixed to obtain good performance and could be adjusted to the exact same settings before model training. This might hamper the accuracy of the model to some extent but improve its robustness simultaneously. However, to minimize the impact of the input data, we did use same reconstruction parameters to reconstruct these SPECT images. Second, the gold standard was set as the diagnostic report made by an agreement of two experienced interpreters because of the dilemma that not every patient received coronary angiography during hospitalization on the one hand, and more MPI images were preferred to be used in the AI training process on the other hand. Third, due to the relatively limited number of lesions in the HLA, both sensitivity and specificity were inevitably lower than other axes in terms of the random validation set. Lower accuracy in some of the segments seen in this study might be partially be addressed by using a larger cohort in the next stage of our study. Last, the extent of ischemia was not included in our training because too many tags require much more samples in the training set. To achieve good accuracy in terms of both segment and extent of ischemia, further larger datasets will surely be required.

## Conclusion

The AI system of our study showed excellent predictive accuracy, agreement, clinical availability, and efficiency in a large, multicenter population with gated-MPI protocols and therefore, might be potentially helpful to aid radiologists in clinical practice and develop more sophisticated models.

## Supplementary Information

**Additional file 1:** **Supplementary Table 1.**  Timeconsumption (seconds) of AI and experienced interpreter.

## Data Availability

All datasets and materials used and/or analyzed during the current study are available from the corresponding authors on any reasonable request.

## Abbreviations

AI: Artificial intelligence
MPI: Myocardial perfusion imaging
CAD: Coronary artery disease
AHA: American heart association
ICA: Invasive coronary angiography
SPECT: Single-photon emission computer tomography
SA: Short-axis
HLA: Horizontal long-axis
VLA: Vertical long-axis
ROI: Region of interest
YOLO: You only look once
SVM: Support vector machine
