# Supplementary material for: Establishment and validation of an AI-aid method in the diagnosis of myocardial perfusion imaging
Source: BMC Med Imaging. 2023 Jun 16;23:84. doi: 10.1186/s12880-023-01037-y (PMC10273563; doi:10.1186/s12880-023-01037-y)
Supplement: Supplementary file 1 — Additional file 1: Supplementary Table 1. Timeconsumption (seconds) of AI and experienced interpreter. [file 12880_2023_1037_MOESM1_ESM.docx]

Supplementary Tab. 1 Time consumption (seconds) of AI and experienced interpreter

|  | Interpreter | Mean | Min | Max | Std | Sum |
| --- | --- | --- | --- | --- | --- | --- |
| SA | AI  Experienced | 27.89  39.14 | 21.28  25.28 | 36.36  59.77 | 4.99  8.55 | 1673.23  2348.67 |
| HLA | AI  Experienced | 28.31  36.05 | 21.04  16.16 | 37.24  55.73 | 5.45  9.04 | 1698.41  2162.89 |
| VLA | AI  Experienced | 28.58  39.22 | 21.16  22.76 | 38.31  63.73 | 6.03  9.05 | 1715.08  2352.98 |

SA, Short axis; HLA, Horizontal long axis; VLA, Vertical long axis
